# CIRBP protects H9C2 cells against myocardial ischemia through inhibition of NF-κB pathway

**DOI:** 10.1590/1414-431X20175861

**Published:** 2017-03-23

**Authors:** T.Y. Long, R. Jing, F. Kuang, L. Huang, Z.X. Qian, T.L. Yang

**Affiliations:** 1 Cardiovascular Department, The Xiangya Hospital of Central South University, Changsha City, Hunan Province, China; 2 Department of Cardiac Surgery, The First Affiliated Hospital of Xiamen University, Xiamen City, Fujian Province, China; 3 Department of Cardiac Surgery, Shenzhen Hospital of Peking University, Shenzhen City, Guangdong Province, China; 4 Department of Emergency, The Xiangya Hospital of Central South University, Changsha City, Hunan Province, China

**Keywords:** Myocardial ischemia, CIRBP, NF-κB, Cell proliferation, Cell apoptosis

## Abstract

Myocardial ischemia is a major cause of death and remains a disease with extremely deficient clinical therapies and a major problem worldwide. Cold inducible RNA-binding protein (CIRBP) is reported to be involved in multiple pathological processes, including myocardial ischemia. However, the molecular mechanisms of myocardial ischemia remain elusive. Here, we first overexpressed CIRBP by transfection of pc-CIRBP (pcDNA3.1 containing coding sequenced for CIRBP) and silenced CIRBP by transfection of small interfering RNA targeting CIRBP (siCIRBP). pcDNA3.1 and the negative control of siCIRBP (siNC) were transfected into H9C2 cells to act as controls. We then constructed a cell model of myocardial ischemia through culturing cells in serum-free medium with hypoxia in H9C2 cells. Subsequently, AlamarBlue assay, flow cytometry and western blot analysis were used, respectively, to assess cell viability, reactive oxygen species (ROS) level and apoptosis, and expression levels of IκBα, p65 and Bcl-3. We demonstrated that CIRBP overexpression promoted cell proliferation (P<0.001), inhibited cell apoptosis (P<0.05), reduced ROS level (P<0.001), down-regulated phosphorylated levels of IκBα and p65 (P<0.01 or P<0.001), and up-regulated expression of Bcl-3 (P<0.001) in H9C2 cells with myocardial ischemia. The influence of CIRBP knockdown yielded opposite results. Our study revealed that CIRBP could protect H9C2 cells against myocardial ischemia through inhibition of NF-κB pathway.

## Introduction

Myocardial ischemia is a major cause of death, leading to a disease for which clinical therapy is extremely deficient worldwide (1). Myocardial ischemia refers to the reduction of blood perfusion in the heart, resulting in reduction of heart oxygenation and abnormal myocardial energy metabolism, so that the normal functioning of the heart cannot be well supported (2,3). Essential to myocardial cell activity, oxygen is transported to cells through blood. The hyperbaric oxygen preconditioning has been proven to be an effective prevention against myocardial infarction (4). The heart relies solely on the myocardial blood supply, so that myocardial ischemia can immediately affect heart function (5–7).

Myocardial ischemia and its related mechanisms, as well as the determinants for this disease, have been partly studied (8,9). Zhang et al. (9) explored the effect of hydrogen sulfide on myocardial ischemia/reperfusion injury (MIRI) and found that hydrogen sulfide could protect against MIRI in mice, and this might be related to the enhancement of antioxidative ability and the decreased release of inflammatory factors. Although great progress has been made in mechanism and therapy research, the biological basis for the increased rate of myocardial ischemia also needs to be deeply understood.

Cold-inducible RNA-binding protein (CIRBP) is a stress response protein found in mammals, which is excessively expressed under cold stress, hypoxia stress, ultraviolet irradiation, etc. (10–13). Studies have shown that CIRBP is expressed in various cell types and is involved in various disease processes, such as cancer, wound healing, sepsis, and others (14–16). CIRBP is constitutively expressed at low levels across several tissues, but the role of CIRBP in myocardial cell hypoxia stress reaction and its underlying mechanism remain deficient.

In this study, we investigated cell viability, apoptosis, and reactive oxygen species (ROS) of H9C2 cells *in vitro*, and confirmed the connection between CIRBP and myocardial ischemia injury. The involved pathway of CIRBP in myocardial ischemia injury was also investigated. The present study provided a new understanding of the regulation of CIRBP in myocardial ischemia injury and identified potential avenues for therapeutic intervention of this disease.

## Material and Methods

### Cell culture and hypoxia treatment

H9C2 cells (Sigma-Aldrich, USA) were maintained in Dulbecco's modified eagle medium (DMEM; Gibco, China) supplemented with 10% fetal bovine serum (FBS; Gibco, USA), 1% penicillin/streptomycin and 1% glutaMAX (both from Life Technologies, USA) at 37°C with 5% CO_2_. For hypoxia treatment, the medium was replaced by serum-free DMEM when the cells reached 80% confluence. Then, cells were incubated in Anaerobe Gas Generating Pouch System (GasPak™ EZ, BD Biosciences, USA) with 10% CO_2_ and 1% O_2_ following the manufacturer’s instructions.

### Plasmids and siRNA transfection

The full-length coding sequence of wild-type CIRBP was sub-cloned into pcDNA3.1 (Invitrogen, USA) to construct pcDNA3.1/CIRBP (pc-CIRBP) which was confirmed by sequencing. The empty pcDNA3.1 was transfected as a control. The target sequence for CIRBP-specific siRNA (siCIRBP; 5′-GUA CGG ACA GAU CUC UGA AdT dT-3′) and its negative control (siNC) were both synthesized by GenePharma (Shanghai, China). Cell transfection was performed in 6-well plates by using Lipofectamine 3000 reagent (Invitrogen, USA). For each well, 2 μg plasmid or 60 pmol siRNA was transfected in line with the manufacturer's protocol.

### AlamarBlue assay

The cell viability was determined using the AlamarBlue assay (Invitrogen, Germany). Briefly, cells (10^4^ cells/well) were seeded onto 96-well plates and cultured under hypoxia for 4 days. Cell viability was assessed daily by replacing the medium with DMEM supplemented with 10% AlamarBlue, followed by incubation for 3 h at 37°C. Thereafter, absorbance was measured at 570 nm (600 nm background subtraction) with a microplate reader (Bio-Rad, USA).

### ROS assay

We measured the ROS levels by flow cytometry using 2,7-dichlorofluorescein diacetate (DCFH-DA) (Jiancheng Bioengineering Institute, China). The cells were cultured in a 6-well plate. After rinsing with phosphate-buffered saline (PBS) twice, cells were co-incubated with serum-free DMEM medium containing 10 μM DCFH-DA for 20 min at 37°C in dark. Then, samples were collected by trypsin digestion method. All samples were centrifuged at 150 *g* for 5 min at 4°C and the supernatants were removed. The cells were resuspended to 500 μL PBS and the fluorescence intensities were measured by using a flow cytometer (488 nm excitation, 521 nm emissions).

### Apoptosis assay

Apoptosis assay was performed by using Annexin V-FITC/PI apoptosis detection kit (Beijing Biosea Biotechnology, China). The adherent and floating cells were combined and washed by pre-cold PBS. Then, cells were resuspended by binding buffer and stained by 10 μL Annexin V-FITC and 5 μL PI in turn, according to the manufacturer's instruction. After that, cells were measured with flow cytometer (Beckman Coulter, USA) to differentiate apoptotic cells (Annexin-V positive and PI-negative) from necrotic cells (Annexin-V and PI-positive).

### Quantitative reverse transcription PCR (qRT-PCR)

Total RNA was isolated from transfected cells by using TRIzol reagent (Invitrogen, USA) and DNaseI (Promega, USA). Reverse transcription was performed by using the MultiScribe reverse transcriptase and a mix of random hexamers and oligo dT (all from Applied Biosystems, USA) at a condition of 10 min at 25°C, 30 min at 48°C and a final step of 5 min at 95°C (17). cDNA was then subjected to amplification by using SYBR Green PCR Master Mix (Applied Biosystem) according to manufacturer's instructions. Relative mRNA expression levels were calculated using the formula 2^-ΔΔCt^ (18). The primers were synthesized as shown in Table 1 (Sangon, China). NAPDH was used as the housekeeping gene.

**Table 1:**
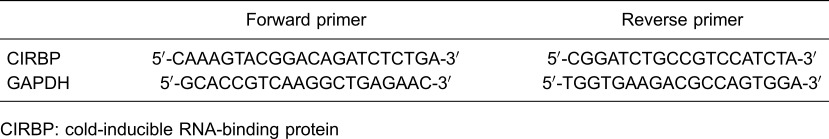
Primer sequences.

### Western blot analysis

The protein used for western blotting was extracted using RIPA lysis buffer (Beyotime Biotechnology, China) supplemented with protease inhibitors (Roche, China) and quantified using the BCA™ Protein Assay Kit (Pierce, USA). The western blot system was established using a Bio-Rad Bis-Tris Gel system according to the manufacturer's instructions. The proteins were separated by 12% sodium dodecyl sulfate-polyacrylamide gel electrophoresis and transferred to polyvinylidene difluoride membrane. Primary antibodies against CIRBP (ab106239), Bcl-3 (ab27780), (both from Abcam, UK); inhibitor of nuclear factor κBα (IκBα, 4812), phosphorylated IκBα (p-IκBα, 5209), p65 (8242), phosphorylated p65 (p-p65, 3031), (all from Cell Signaling Technology, USA) and GAPDH (G9545, Sigma, USA) were diluted and incubated with the membrane at 4°C overnight, followed by wash and incubation with secondary antibodies marked by horseradish peroxidase (HRP) for 1 h at room temperature. After rinsing, the membranes with blots and antibodies were transferred into the Bio-Rad ChemiDoc™ XRS system, followed by addition of 200 μL Immobilon Western Chemiluminescent HRP Substrate (Millipore, USA) to cover the membrane surface. The signals were captured and the intensity of the bands was quantified using Image Lab™ Software (Bio-Rad).

### Statistical analysis

Data are reported as means±SD. Statistical analyses were performed using Graphpad statistical software (GraphPad, USA). The P values were calculated using one-way analysis of variance (ANOVA). A P value of <0.05 indicated a statistically significant result.

## Results

### pc-CIRBP and siCIRBP successfully interfered with the expression of CIRBP

**Figure 1:**
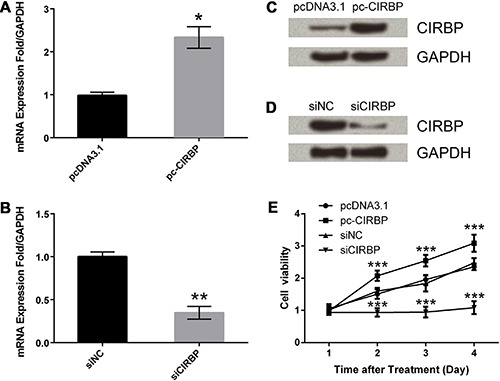
Effects of cold-inducible RNA-binding protein (CIRBP) on cell viability of H9C2 cells with myocardial ischemia. CIRBP mRNA expression levels in cells transfected with pcDNA3.1 or pc-CIRBP (*A*) and siNC or siCIRBP (*B*) were assessed by quantitative reverse transcription PCR. CIRBP protein expression levels in cells transfected with pcDNA3.1 or pc-CIRBP (*C*) and siNC or siCIRBP (*D*) were assessed by western blot analysis. (*E*), Cell viability of transfected cells. Cells were transfected with pcDNA3.1, pc-CIRBP, siNC or siCIRBP and cultured in serum-free medium with hypoxia. Cell viability was determined by AlamarBlue assay. pc-CIRBP: pcDNA3.1 containing CIRBP coding sequence; siCIRBP: CIRBP-specific small interfering RNA; siNC: siCIRBP negative control. Data are reported as means±SD of 3 independent experiments. *P<0.05, **P<0.01, ***P<0.001 (ANOVA).

H9C2 cells were transfected with siCIRBP, pc-CIRBP and their controls, respectively. qRT-PCR and western blot analysis displayed the expression levels of CIRBP at both mRNA and protein levels. As shown in Figure 1A and C, the CIRBP expression levels were significantly increased by pc-CIRBP when compared to cells transfected with pcDNA3.1 (P<0.05). At the same time, the CIRBP expression levels were markedly decreased by siCIRBP compared with cells transfected with negative control of siCIRBP (siNC) (Figure 1B and D, P<0.01). Thus, we concluded that the transfection of pc-CIRBP successfully overexpressed CIRBP while transfection of siCIRBP successfully silenced CIRBP.

### CIRBP promoted cell proliferation

To investigate the effects of CIRBP on cell proliferation, the AlamarBlue assay was employed to test the cell viability of myocardial ischemia cells. In Figure 1E, overexpression of CIRBP significantly enhanced cell viability at 2-4 days compared with its control (P<0.001). Meanwhile, CIRBP knockdown significantly reduced cell viability at 2-4 days compared with its control (P<0.001). Thus, we confirmed that CIRBP could promote cell proliferation in myocardial ischemia cells.

### CIRBP suppressed cell apoptosis

**Figure 2:**
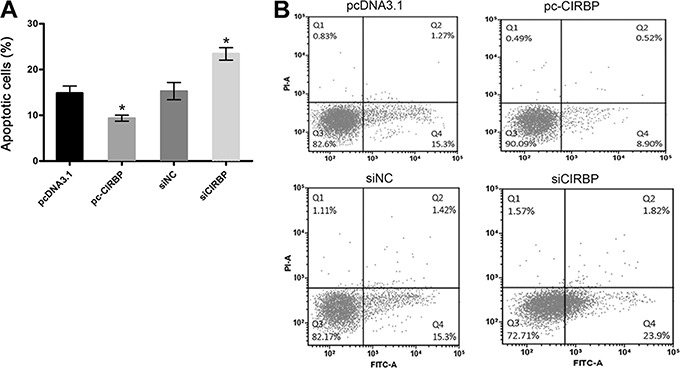
Effects of cold-inducible RNA-binding protein (CIRBP) on cell apoptosis of H9C2 cells with myocardial ischemia. Cells were transfected with pcDNA3.1, pc-CIRBP, siNC or siCIRBP and cultured in serum-free medium with hypoxia. Cell apoptosis was evaluated by flow cytometry. pc-CIRBP: pcDNA3.1 containing CIRBP coding sequence; siCIRBP: CIRBP-specific small interfering RNA; siNC: siCIRBP negative control. Data are reported as means±SD of 3 independent experiments. *P<0.05, pc-CIRPB compared to pcDNA3.1 and siCIRBP compared to siNC (ANOVA).

To investigate the effects of CIRBP on cell apoptosis, flow cytometry was employed to assess cell apoptosis of myocardial ischemia cells. The results in Figure 2 showed that overexpression of CIRBP inhibited cell apoptosis compared with its control (P<0.05), while CIRBP knockdown promoted cell apoptosis compared with its control (P<0.05). Thus, we concluded that CIRBP could inhibit cell apoptosis in myocardial ischemia cells.

### CIRBP reduced ROS levels

**Figure 3:**
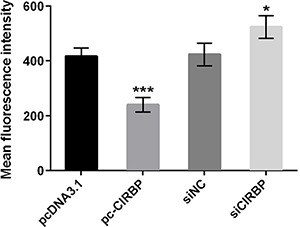
Effects of cold-inducible RNA-binding protein (CIRBP) on reactive oxygen species (ROS) levels of H9C2 cells with myocardial ischemia. Cells were transfected with pcDNA3.1, pc-CIRBP, siNC or siCIRBP and cultured in serum-free medium with hypoxia. The ROS level was evaluated by flow cytometry using DCFH-DA. pc-CIRBP: pcDNA3.1 containing CIRBP coding sequence; siCIRBP: CIRBP-specific small interfering RNA; siNC: siCIRBP negative control; DCFH-DA: 2,7-dichlorofluorescein diacetate. Data are reported as means±SD of 3 independent experiments. *P<0.05, siCIRBP compared to siNC; ***P<0.001, pc-CIRPB compared to pcDNA3.1 (ANOVA).

We measured ROS levels by flow cytometry. The ROS levels in Figure 3 clearly showed that overexpression of CIRBP significantly reduced ROS levels compared with its control (P<0.001), while knockdown of CIRBP enhanced ROS levels significantly compared with its control (P<0.05). Therefore, CIRBP could obviously reduce ROS levels.

### Effect of CIRBP on myocardial ischemia was related to NF-κB pathway

**Figure 4:**
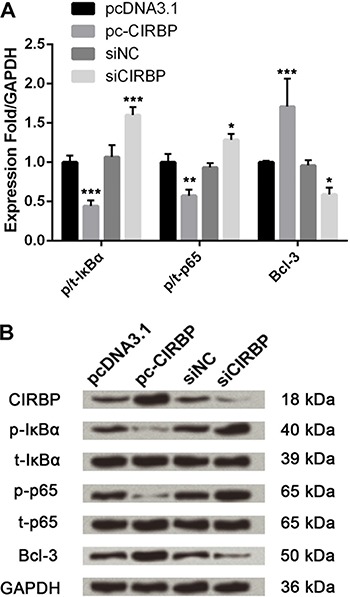
Effects of cold-inducible RNA-binding protein (CIRBP) on NF-κB signaling pathway. Cells were transfected with pcDNA3.1, pc-CIRBP, siNC or siCIRBP and cultured in serum-free medium with hypoxia. Protein expression was evaluated by western blot analysis. The band intensity was assessed by Image Lab™ software. The phosphorylation rate is reported as the relative intensity of phosphorylated kinases/total kinases and the final results were normalized by GAPDH. pc-CIRBP: pcDNA3.1 containing CIRBP coding sequence; siCIRBP: CIRBP-specific small interfering RNA; siNC: siCIRBP negative control; IκBα: inhibitor of nuclear factor κBα; p-IκBα: phosphorylated IκBα; p-p65: phosphorylated p65. Data are reported as means±SD of 3 independent experiments. *P<0.05, **P<0.01, ***P<0.001, pc-CIRPB compared to pcDNA3.1 and siCIRBP compared to siNC (ANOVA).

Based on the above results, we focused on the nuclear factor-κB (NF-κB) pathway. As shown in Figure 4, phosphorylated levels of IκBα and p65 were markedly down-regulated (P<0.01 or P<0.001) but expression level of Bcl-3 was up-regulated by CIRBP overexpression compared to their controls. Meanwhile, the influence of CIRBP knockdown was opposite to CIPRB overexpression, resulting in up-regulation of IκBα and p65 phosphorylation and down-regulation of Bcl-3 expression (P<0.05 or P<0.001). Thus, the effects of CIRBP on myocardial ischemia could be connected with the NF-κB pathway activation.

## Discussion

Myocardial ischemia remains the leading cause of morbidity and mortality despite of significant progress in cardiovascular medicine (19). Results suggest that many factors influence the progress and development of myocardial ischemia (20,21). CIRBP, which is the first cold-shock protein identified in mammals, was recently identified as a proinflammatory cytokine (22). As a sensor protein, which expression increases in response to stress, CIRBP plays important roles in tumor recurrence (23,24). A study by Zhang et al. (25) has shown that CIRBP plays key roles in hypoxia-induced cell cycle arrest and may be utilized for preventing hypoxia-induced neonatal brain injury. Therefore, in our study, we focused on CIRBP and explored its role in transfected H9C2 cells. Myocardial ischemia is a process that causes reduced blood supply to the heart, resulting in simultaneous nutrient and oxygen deprivation (26). A vast number of studies used H9C2 cells to simulate myocardial ischemia for addressing MIRI (27,28).

The results implied that cell proliferation was promoted by CIRBP overexpression but suppressed by CIRBP knockdown. At the same time, the effect of CIRBP on cell apoptosis was opposite to proliferation. The results in our study are in agreement with previous investigations. Jian et al. suggested that CIRBP up-regulation could induce corticotroph cell proliferation in pituitary corticotroph adenoma (29). Li et al. (30) has demonstrated that CIRBP inhibited H_2_O_2_-induced apoptosis in rat cortical neurons. However, another study interestingly found that CIRBP significantly activated inflammasome and thereby induced cell death of mouse lung vascular endothelial cell (11 [Bibr B12]). Thus, we suspected that the specific influence of CIRBP on cell proliferation and apoptosis varied depending on cell types. Our study was the first to explore the role of CIRBP in myocardial cells. Studies on CIRBP as a therapeutic drug should continue to support the application of CIRBP on clinical therapy of myocardial ischemia.

Studies have reported that starvation and hypoxia frequently trigger production of ROS, which is shown to largely damage cardiomyocyte (31,32). Massive ROS production causes cell death due to necrosis. In the present study, the ROS level in cells with myocardial ischemia was significantly reduced by CIRBP overexpression while markedly enhanced by CIRBP knockdown. The alteration of ROS levels could partially explain the effect of CIRBP on cell proliferation and apoptosis.

Overproduction of ROS leads to oxidative stress, and NF-κB is an essential transcription factor, which could be activated by oxidative stress and effectively regulate cell apoptosis (33). Thus, we speculated that the influence of CIRBP on cell proliferation and apoptosis might be associated with NF-κB signaling pathway. IκBα, an effective inhibitor of NF-κB, is located in the cytoplasm and binds to NF-κB (p50-p65 heterodimer) in the unstimulated cells (34). Under oxidative stress, the IκBα could be phosphorylated, ubiquitinated and degraded, followed by phosphorylation of p65 (35). The phosphorylated p65 then translocates to the nucleus and binds to a specific sequence, resulting in gene transcription including pro-apoptotic genes (36). In our study, the phosphorylated levels of IκBα and p65 were both down-regulated by CIRBP overexpression, which might lead to repression of cell apoptosis. In terms of Bcl-3, accumulating experiments demonstrate its role as proliferative factor (37). On the one hand, Bcl-3 could bind to NF-κB (p50-p52 heterodimer) and then activate cyclin-D1 promoter, resulting in increased cell proliferation (38). On the other hand, Bcl-3 is implied to block apoptosis in IL-4-deprived cells (39). In our study, the expression level of Bcl-3 was markedly up-regulated by CIRBP overexpression, which might be another explanation for the effect of CIRBP on proliferation and apoptosis of H9C2 cells with myocardial ischemia.

Our study found that overexpression of CIRBP promoted cell proliferation and suppressed cell apoptosis via the NF-κB signaling pathway. This paper preliminarily studied the role of CIRBP in myocardial ischemia injury as well as the underling mechanism, which might provide a theoretical basis for the treatment of myocardial ischemia injury.
